# Supplementary material for: Specific Cell Targeting by Toxoplasma gondii Displaying Functional Single-Chain Variable Fragment as a Novel Strategy; A Proof of Principle
Source: Cells. 2024 Jun 4;13(11):975. doi: 10.3390/cells13110975 (PMC11172386; doi:10.3390/cells13110975)
Supplement: Supplementary file 1 [file cells-13-00975-s001.zip › cells-2995460-supplementary.pdf]

File S1

CAT GFP expression cassette

Cassette, bordered by LoxP sites, to express the fusion protein CAT-GFP driven by the promoter of the *T gondii* α -tubulin gene (αTUB) and the 3 'untranslated region of the *T. gondii* SAG1 gene (3'UTR SAG1)

Color code :

HindIII LoxP PromotorTUB CAT AvrII GFP 3'UTR SAG1 loxP XbaI

AAGCTTATAACTTCGTATAATGTATGCTATACGAAGTTATGATATGCATGTCCGCGTTCGTGAAATCTCTGATC
AAGCGGAGTGATCAGGAATCATCGTCTCAGCGGGATGACGTTGCGGAGCAGGCCGGCTCGCGGTGGGCAG
TCAGATGCCGAAGGCGTAACTCAGGACGGCTTGCGCTCATCGCAGAACAGGGGTGGTGCCTGCATTGGGTG
CGGTTGGTGATCCTGGTTGGACCGGTGGAGATGCGCGCGCACGAAGGGGATGTGTCAGAAACATTTTGTTT
GTTCTCTGTGAACTTTTAGATGTGTTAAAGGCGGCGAATATTAGCAGAGAGTCCTCCTTGTTGGATTCTCTCT
TGAATTTCGCCCTTTCCTTCTCTTTGCGAGTCTCGTAGAGAACAAGCACTCGTTCGCCGTCCCTGACGACGC
AACCCGCGCAGAAGACATCCACCAAACGGTGTTACACAATCACCTTGTGTGAAGTTCTTGCGGAAAACTACT
CGTTGGCATTTTTTCTTGAATTCCCTTTTTCGACAAA**ATG**CATGAGAAAAAAATCACTGGATATACCACCGTTG
ATATATCCCAATGGCATCGTAAAGAACATTTTGAGGCATTTCAGTCAGTTGCTCAATGTACCTATAACCAGAC
CGTTCAGCTGGATATTACGGCCTTTTTAAAGACCGTAAAGAAAAATAAGCACAAGTTTTATCCGGCCTTTATTC
ACATTCTTGCCCGCCTGATGAATGCTCATCCGGAATTCCGTATGGCAATGAAAGACGGTGAGCTGGTGATAT
GGGATAGTGTTCACCCTTGTTACACCGTTTTCCATGAGCAAACTGAAACGTTTTCATCGCTCTGGAGTGAATA
CCACGACGATTTCCGGCAGTTTCTACACATATATTCGCAAGATGTGGCGTGTTACGGTGAAAACCTGGCCTA
TTTCCCTAAAGGGTTTATTGAGAATATGTTTTTCGTCTCAGCCAATCCCTGGGTGAGTTTCACCAGTTTTGATT
TAAACGTGGCCAATATGGACAACTTCTTCGCCCCCGTTTTCACCATGGGCAAATATTATACGCAAGGCGACAA
GGTGCTGATGCCGCTGGCGATTCAGGTTCATCATGCCGTTTGTGATGGCTTCCATGTCGGCAGAATGCTTAA
TGAATTACAACAGTACTGCGATGAGTGGCAGGGCGGGGCTCCTAGGATGGTGAGCAAGGGCGAGGAGCTG
TTCACCGGGGTGGTGCCCATCCTGGTCGAGCTGGACGGCGACGTAAACGGCCACAAGTTCAGCGTGTCCG
GCGAGGGCGAGGGCGATGCCACCTACGGCAAGCTGACCCTGAAGTTCATCTGCACCACCGGCAAGCTGCC
CGTGCCCTGGCCCACCCTCGTGACCACCCTGACCTACGGCGTGCAGTGCTTCAGCCGCTACCCCGACCACA
TGAAGCAGCACGACTTCTTCAAGTCCGCCATGCCCGAAGGCTACGTCCAGGAGCGCACCATCTTCTTCAAG
GACGACGGCAACTACAAGACCCGCGCCGAGGTGAAGTTCGAGGGCGACACCCTGGTGAACCGCATCGAGC
TGAAGGGCATCGACTTCAAGGAGGACGGCAACATCCTGGGGCACAAGCTGGAGTACAACTACAACAGCCAC
AACGTCTATATCATGGCCGACAAGCAGAAGAACGGCATCAAGGTGAACTTCAAGATCCGCCACAACATCGAG
GACGGCAGCGTGCAGCTCGCCGACCACTACCAGCAGAACACCCCCATCGGCGACGGCCCCGTGCTGCTGC
CCGACAACCACTACCTGAGCACCCAGTCCGCCCTGAGCAAAGACCCCAACGAGAAGCGCGATCACATGGTC
CTGCTGGAGTTCGTGACCGCCGCCGGGATCACTCTCGGCATGGACGAGCTGTACAAGTACTCAGATCTCGA
GCTCGCTAGGAATGCATTAGAGGTCCACACGAACCAGGACCCGCTCGATTAAT**TAA**TCACCGTTGTGCTCAC
TTCTCAAATCGACAAAGGAAACACACTTCGTGCAGCATGTGCCCCATTATAAAGAAACTGAGTTGTTCCGTTG
TGGCTTGCAGGTGTCACATCCACAGAAACCGGCCGACTCTAAATAGGAGTGTTTCGCAGCAAGCAGCGAAA
GTTTATGACTGGGTCCGAATCTCTGAACGGATGTGTGGCGGACCTGGCTGATGTTGATCGCCGTCGACACA
CGCGCCACATGGGTCAATACACAAGACAGCTATCAGTTGTTCTAGTCGAACCGGTTAACACAATTCTTGCCC
CCCCGAGGGATAACTTCGTATAATGTATGCTATACGAAGTTATGGATCCACTAGTTCTAGA

Expression cassette

Cassette to express a protein of interest driven by a modified promoter of the *T gondii* α - tubulin gene (αTUB8) and the 3 ' untranslated region of the *T. gondii* SAG1 gene (3'UTR SAG1)

Length: 1018 bp
5 repeat motifs underlined (Soldati et al, 1995)

Promotor αTUB8: XbaI/PmeI (509 bp)
3'UTR SAG1 : NotI/AvrII (345 bp)
Cloning sites : PmeI (510), NotI (532)

TCTAGAAGTATACTATTCGAAAAACTAGTATCGATAAGCTTGAACACGCGTCTCAGGGTGCGTGG
TTAGAACACGCGTCTCAGGGTGCGTGGTTAGAACACGCGTCTCAGGGTGCGTGGTTGGAACACG
CGTGTCAGGGTGCGTGGTTGGAACACGCGTCTCAAGGTTTGTGGTTAAGCTTGCCTGCATTGGG
TGCGGTTGGTGATCCTGGTTGGACCGGTGGAGATGCGCGCGCACGAAGGGGATGTGTCAGAAA
CATTTTGTTTGTTCTCTGTGAACTTTTAGATGTGTTAAAGGCGGCGAATATTAGCAGAGAGTCCTC
CTTGTTGGATTCTCTCTTGAATTTCGCCCTTTCCTTCTCTTTGCGAGTCTCGTAGAGAACAAGCAC
TCGTTCGCCGTCCCTGACGACGCAACCCGCGCAGAAGACATCCACCAAACGGTGTTACACAATCA
CCTTGTGTGAAGTTCTTGCGGAAAACTACTCGTTGGCATTTTTTCTTGAATTCCCTGTTTAAACAAA
ATTCTTAAGTTGCGGCCGCTTCCATGGTTTTAATTAATCACCGTTGTGCTCACTTCTCAAATCGAC
AAAGGAAACACACTTCGTGCAGCATGTGCCCCATTATAAAGAAACTGAGTTGTTCCGCTGTGGCT
TGCAGGTGTCACATCCACAAAAACCGGCCGACTCTAAATAGGAGTGTTTCGCAGCAAGCAGCGA
AAGTTTATGACTGGGTCCGAATCTCTGAACGGATGTGTGGCGGACCTGGCTGATGTTGATCGCC
GTCGACACACGCGCCACATGGGTCAATACACAAGACAGCTATCAGTTGTTTTAGTCGAACCGGTT
AACACAATTCTTGCCCCCCCGAGGCGCGCCAACCTAGGAGGGCCCGGGCGCCATGGCAGCTGA
CGTCTTATGCTTCTGAGATATCTTCAAAAGATATATAGCTAATGGCGTATAAGATTATTTAAAATAA
ATTTTTAGCCAAACTGTCTCACGACGTTTTGAACCCAGTCTAGA

XbaI PmeI NotI AvrII

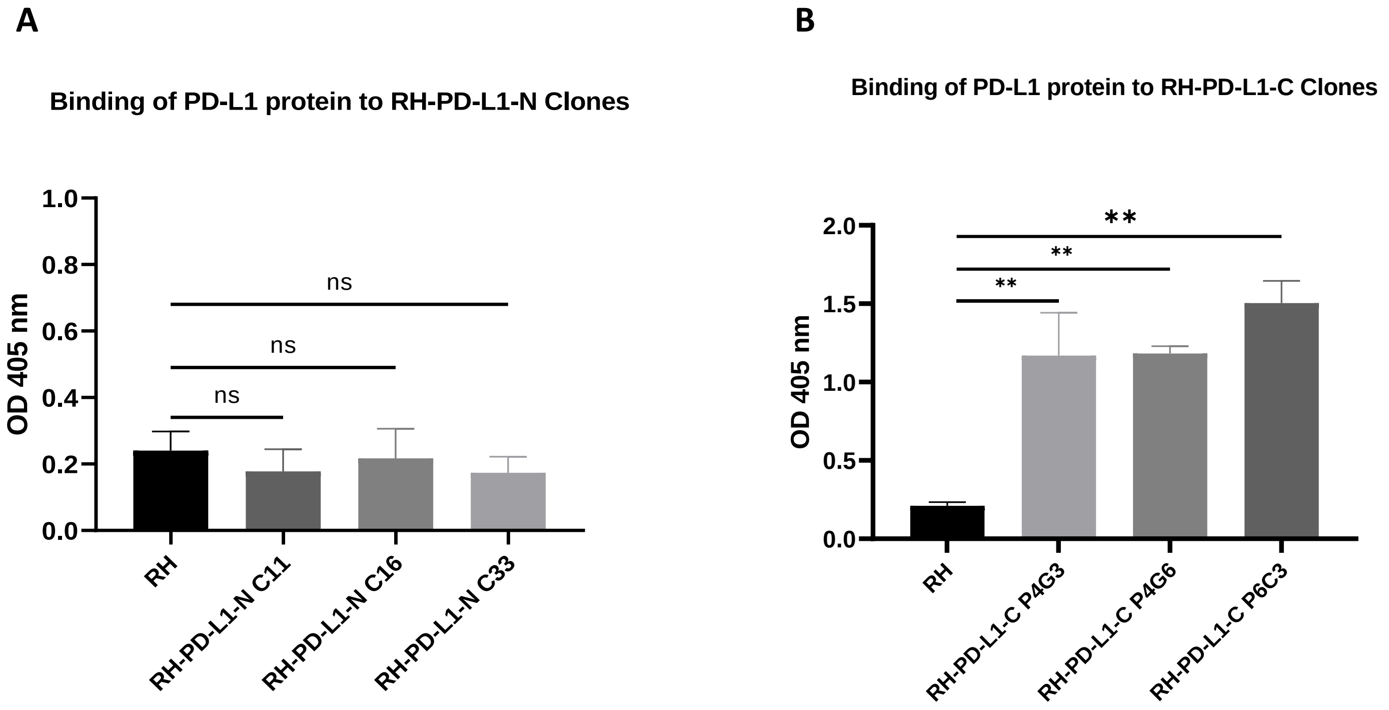

Figure S1. Binding of RH-PD-L1-N and RH-PD-L1-C clones to PD-L1 protein. Tachyzoites of the selected clones of RH-PD-L1-N **(A)** and RH-PD-L1-C **(B)** recombinant strains were incubated with histidine-tagged PD-L1 recombinant protein and bound protein was assessed by ELISA using anti-His tag antibody (n= 3 replicates). Optical densities were read at 405 nm. Statistical significance is indicated by **$p < 0.01$. ns: not significant. All experiments were performed at least three times.

A

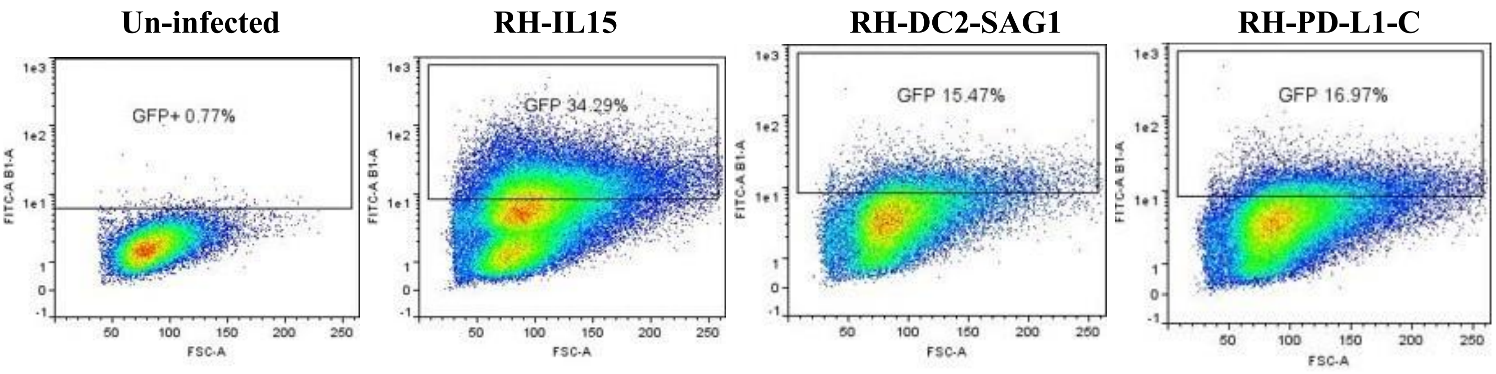

B

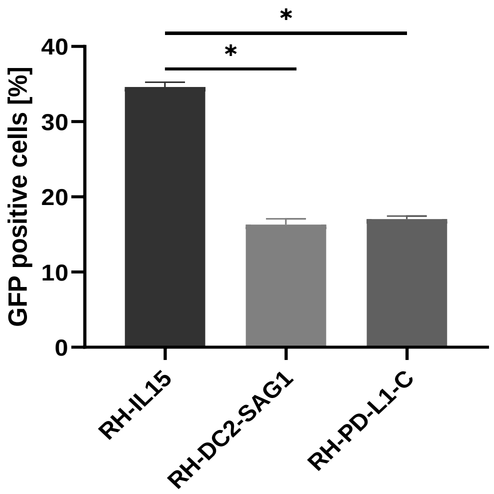

Figure S2. Replication of RH-PD-L1-C and RH-DC2-SAG1 recombinant tachyzoites in non-targeted cells. The replication of RH-PD-L1-C and RH-DC2-SAG1 tachyzoites in HFF cells was compared to the recombinant RH strain engineered to secret IL-15 (as a surface-non-modified, GPF positive strain). HFF cells were infected with each of the indicated strain at MOI 1, then, incubated for 1 h at 37 °C to allow for invasion. Following incubation, cells washed to remove extracellular tachyzoites and incubated for 24 h. Invasion was analyzed by flow cytometry. **(A)** Representative flow cytometry dot blots showing the infected cells indicated by the GFP positive cells. **(B)** Quantification of invasion in 3 replicates. Statistical significance is indicated by *$p < 0.05$. Experiments was performed three times.
